# Associations Between Polyvascular Disease and Stroke Recurrence in Patients With Lacunar Stroke

**DOI:** 10.3389/fneur.2021.706991

**Published:** 2021-09-08

**Authors:** Lin Ma, Anxin Wang, Yijun Zhang, Yilong Wang, Yongjun Wang, Xia Meng

**Affiliations:** ^1^Department of Neurology, Beijing Tiantan Hospital, Capital Medical University, Beijing, China; ^2^Department of Neurology, China National Clinical Research Center for Neurological Diseases (NCRC-ND), Beijing, China; ^3^Advanced Innovation Center for Human Brain Projection, Capital Medical University, Beijing, China

**Keywords:** stroke, polyvascular disease, small vessel occlusion, cornary heart disease, peripheral artery (occlusive) disease

## Abstract

**Background and purpose:** This study aimed to examine the association of polyvascular disease and clinical outcomes in patients with lacunar stroke.

**Methods:** Data of patients with recent lacunar stroke were collected from The Third China National Stroke Registry. Polyvascular disease is defined as the existence of atherosclerosis across two or more vascular beds. For the present study, polyvascular disease patients were grouped as follows: coronary heart disease (CHD) and lacunar stroke, peripheral arterial disease (PAD) and lacunar stroke, and CHD/PAD and lacunar stroke. The major clinical outcome was recurrence, and the secondary clinical outcome was major adverse cardiovascular events (MACEs). A Cox proportional multivariable hazards regression model was applied to estimate the association between polyvascular disease and outcomes.

**Results:** Among 3,165 patients with recent lacunar stroke, CHD was present in 375 (11.8%) and peripheral arterial disease in 168 (5.3%). The hazard ratio (HR) for stroke recurrence was 0.98 (95% confidence interval [CI], 0.65–1.46; *p* = 0.91) for patients with CHD and lacunar stroke, 1.07 (95% CI, 0.61–1.87; *p* = 0.82) for patients with PAD and lacunar stroke, and 0.95 (95% CI, 0.66–1.35; *p* = 0.75) for patients with CHD/PAD and lacunar stroke compared with patients with isolated lacunar stroke. The HR for MACEs was 1.01 (95% CI, 0.69–1.49; *p* = 0.94) for patients with CHD and lacunar stroke, 1.11 (95% CI, 0.65–1.90; *p* = 0.71) for patients with PAD and lacunar stroke, and 0.99 (95% CI, 0.70–1.40; *p* = 0.95) for patients with CHD/PAD and lacunar stroke.

**Conclusion:** Polyvascular disease is not associated with recurrence of stroke and MACEs in patients with recent lacunar stroke at 1 year.

## Introduction

Atherosclerosis is a systemic disease, which mainly affects the large and middle arteries. The major clinical manifestations of atherosclerosis include coronary heart disease (CHD), peripheral arterial disease (PAD), and ischemic stroke (1). Polyvascular disease is defined as the existence of atherosclerosis across two or more vascular beds. Previous studies have shown that polyvascular disease has great value in predicting the risk of ischemic events (2–6). A secondary analysis of the international Examining Use of Ticagrelor in Peripheral Artery Disease (EUCLID) trial reported that concomitant polyvascular disease was associated with increased long-term cardiovascular risk in patients with CHD (5). Furthermore, in patients with PAD, the risk of major adverse cardiovascular events (MACEs) increases with damage to each additional vascular bed (5).

However, the lacunar stroke is mainly caused by pathological changes in cerebral small vessels. The characteristics and risk factors in cerebral small vessels are different from those in intracranial/extracranial large arteries, coronary arteries, and peripheral arteries (7, 8), and treatment of cerebral small vessel disease is also different (9). Investigating the association between polyvascular diseases and lacunar stroke is important. Little is known regarding whether polyvascular disease increases the risk of ischemic events in patients with lacunar stroke. Therefore, we aimed to assess the prevalence of polyvascular disease in patients with lacunar stroke, and to determine the risk of stroke and MACEs in patients with lacunar stroke.

## Methods

### Study Population

The Third China National Stroke Registry (CNSR-III) is a nationwide prospective registry for patients who present to hospital with acute ischemic stroke. The CNSR-III study collected data on 15 166 patients with acute ischemic stroke from August 2015 to March 2018. All participants were Chinese. Inclusion criteria were: (1) age older than 18; (2) Ischaemic stroke or TIA; (3) Within 7 days from the onset of symptoms to enrolment; (4) Informed consent from patient or legally authorized representative (primarily spouse, parents, adult children, otherwise indicated). Patients who had silent cerebral infarction with no manifestation of symptoms and signs or who refused to participate in the registry were excluded. Acute ischaemic stroke was diagnosed according to the WHO criteria and confirmed by MRI or brain CT. Details of the design, rationale, and major results have been previously described (10). The CNSR-III study protocol was approved by the ethics committee of Beijing Tiantan Hospital. Written informed consent was obtained from all participants or their legal representatives before entering the study.

**Figure 1:**
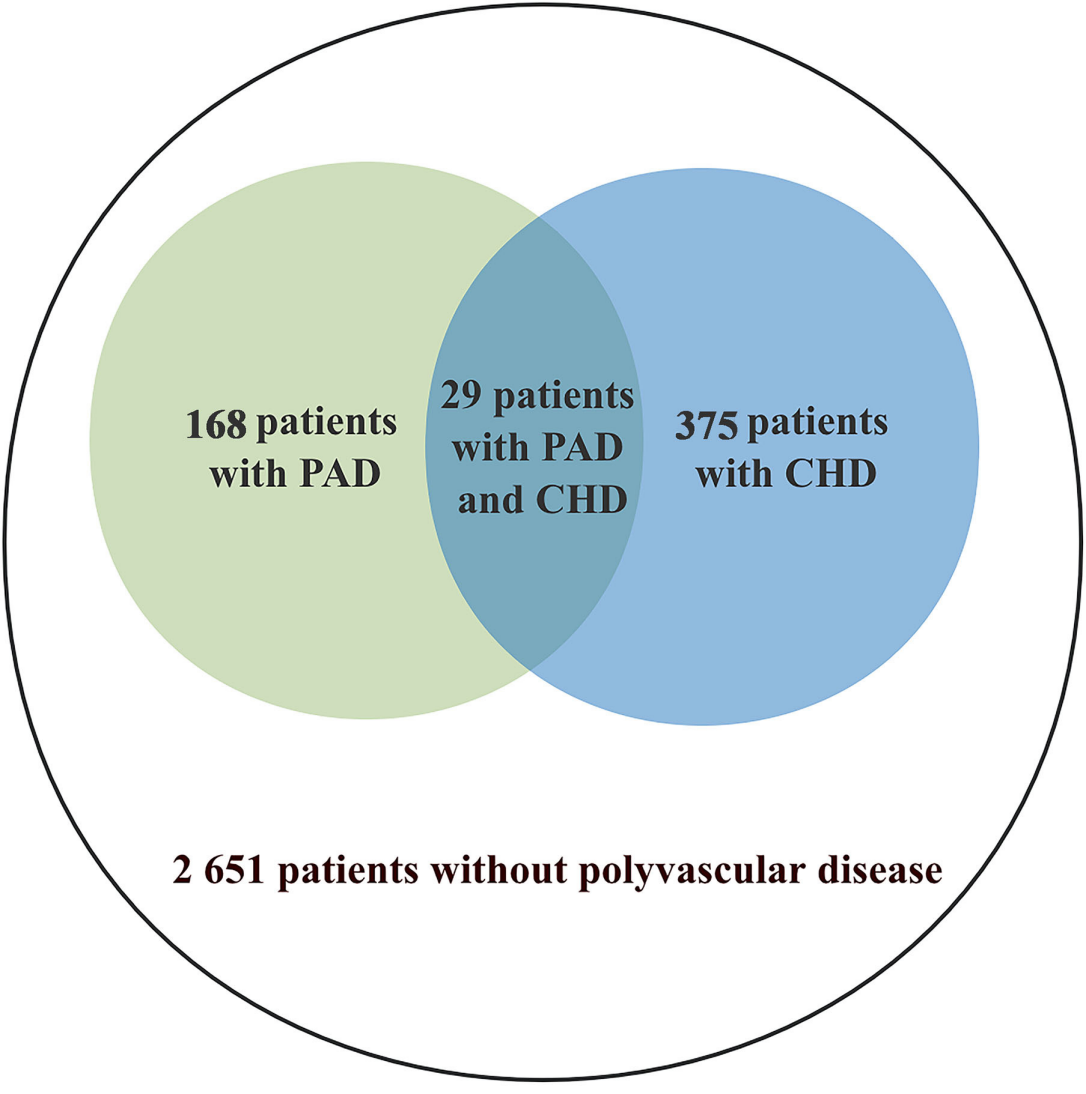
Study population and subgroups.

Recent lacunar stroke was determined by the presence of a single relevant brain stem or subcortical hemispheric lesion, which was <1.5 cm in diameter, with increased signal on diffusion-weighted imaging, reduced signal on apparent diffusion coefficient map, increased signal on fluid attenuated inversion recovery (FLAIR), increased T2-weighted imaging, reduced signal on T1-weighted MRI (11). The imaging data were evaluated by neurologists who were trained in image interpretation. Data of the present study were derived from the recent lacunar stroke subgroup of the CNSR-III. A total of 3,165 patients with recent lacunar stroke voluntarily participated in the present study (Figure 1).

In this study, patients were grouped by the final diagnosis of hospitalization as follows: CHD/PAD and lacunar stroke, CHD and lacunar stroke, PAD and lacunar stroke, and lacunar stroke alone. The group of CHD/PAD and lacunar stroke was composed of patients with lacunar stroke had CHD or PAD. The definition of CHD was a history of CHD that led to percutaneous coronary intervention, coronary artery bypass grafting with revascularization, or objective evidence of coronary artery disease (≥50% stenosis) in at least two arteries (12). PAD was defined as intermittent claudication and an ankle brachial index < 0.90 obtained in the previous 12 months, prior peripheral revascularization, or amputation of the legs at any level due to arterial obstructive disease (13).

### Assessment of Outcomes

This study's primary outcome was recurrence of stroke (including ischemic or hemorrhagic stroke) at 1 year ± 7 days. Recurrence of stroke was adjudicated by a clinical events committee of neurological specialists, while clinical manifestations or imaging was consistent with the diagnosis of stroke. Secondary outcomes were MACEs at 1 year ± 7 days. MACEs were defined as cardiovascular death, myocardial infarction (MI), and ischemic stroke (14, 15). In this study, MI was defined as a rise and fall of cardiac biomarkers (preferably troponin) combined with at least one clinical feature, such as symptoms of ischemic stroke lasting >30 min, changes in an electrocardiogram, during ischemia, pathological Q waves, echocardiography, and invasive coronary angiography, according to the third universal MI definition (16).

Safety outcomes included the occurrence of major bleeding events, as reported by the site of bleeding. Major bleeding was defined as any of the following: intracranial hemorrhage, a witnessed retroperitoneal bleeding event, an absolute drop in hematocrit of ≥12%, baseline hematocrit ≥28% and red blood cell transfusion.

### Statistical Analysis

Continuous variables are presented as mean with standard deviation or median with interquartile range. Comparison of baseline characteristics among the included and excluded patients was made using the non-parametric Kruskal–Wallis test. Categorical variables are reported as counts and percentages and were compared using the χ^2^ test for categorical variables.

A Cox proportional multivariable hazards regression model was applied to estimate the association between polyvascular disease and outcomes. Differences in the rates of stroke, MACEs, and bleeding events during the 1-year follow up are shown as hazard ratios (HRs) and 95% confidence intervals (95% CIs). The model used in this study was adjusted for the following variables: age, sex, education, smoking, alcohol drinking, medical history of ischemic stroke, atrial fibrillation, hypertension, diabetes, hyperlipidemia, National Institutes of Health Stroke Scale score at admission, and stroke type. The level of significance was *p* < 0.05 (two-sided).

All analyses were performed with SAS 9.4 (SAS Institute Inc., Cary, NC). Data are available to researchers for reproducing the results or replicating the procedures by contacting the corresponding author.

## Results

### Baseline Characteristics

A total of 3,165 patients were included in this study. Polyvascular disease was present in 16.2% of the patients: 375 patients (11.8%) had CHD as indicated by the final diagnosis of hospitalization, 168 (5.3%) had PAD, 514 (16.2%) had PAD or CHD. The baseline variables were balanced in patients who were included and excluded from this study (Table 1). The median age of the participants included in the study was 61 years and 28.8% of them were women. A total of 2,056 (65.0%) patients had a history of hypertension and 761 (24.0%) had a history of diabetes.

**Table 1:** Baseline characteristics of patients with lacunar stroke vs. those with other types of stroke.

**Characteristic**	**Included (*n* = 3,165)**	**Excluded (*n* = 12,001)**	***p-Value***
Age (years)	61.1 ± 10.6	62.5 ± 11.5	<0.001
Median	61.0	63.0	
Interquartile range	53.0–68.0	54.0–71.0	
Female sex, no. (%)	911 (28.78)	3,891 (32.42)	<0.001
Medical history, no. (%)			
Hypertension	2,056 (64.96)	7, 438 (61.98)	0.002
Dyslipidemia	240 (7.58)	951 (7.92)	0.55
Diabetes mellitus	761 (24.04)	2,749 (22.91)	0.18
Ischemic stroke	625 (19.75)	2,524 (21.03)	0.12
TIA	48 (1.52)	368 (3.07)	<0.001
Coronary artery disease	229 (7.24)	1,379 (11.49)	<0.001
Known atrial fibrillation	0 (0.00)	1,019 (8.49)	<0.001
Flutter valvular heart disease	1 (0.43)	59 (3.12)	0.01
Current Smoking, no. (%)	1,120 (35.39)	3, 632 (30.26)	<0.001

*TIA, Transient Ischemic Attacks*.

### Association Between Polyvascular Disease and Stroke Recurrence

**Figure 2:**
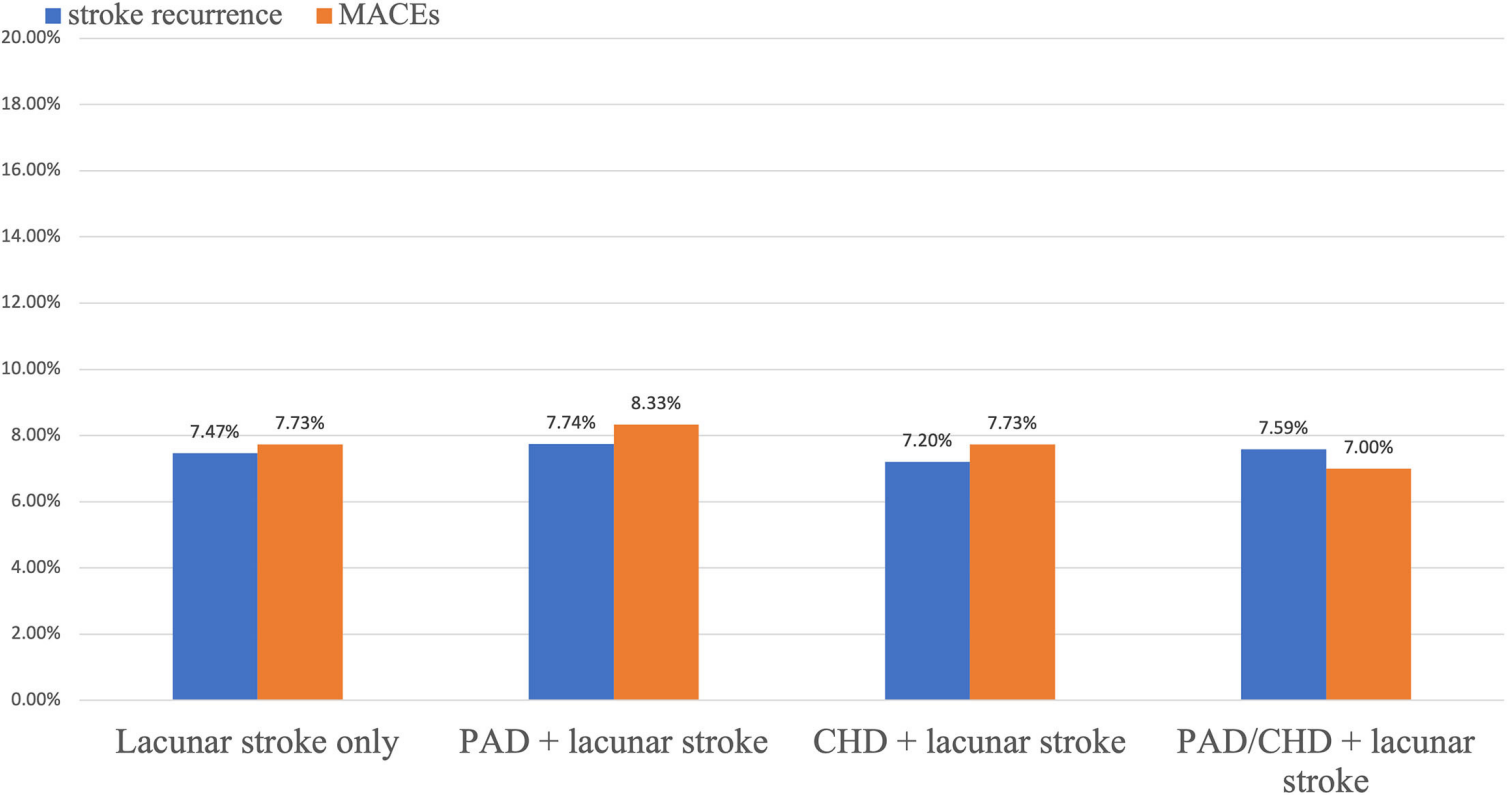
Rates of stroke recurrence and MACEs in patients with recent lacunar stroke.

**Figure 3:**
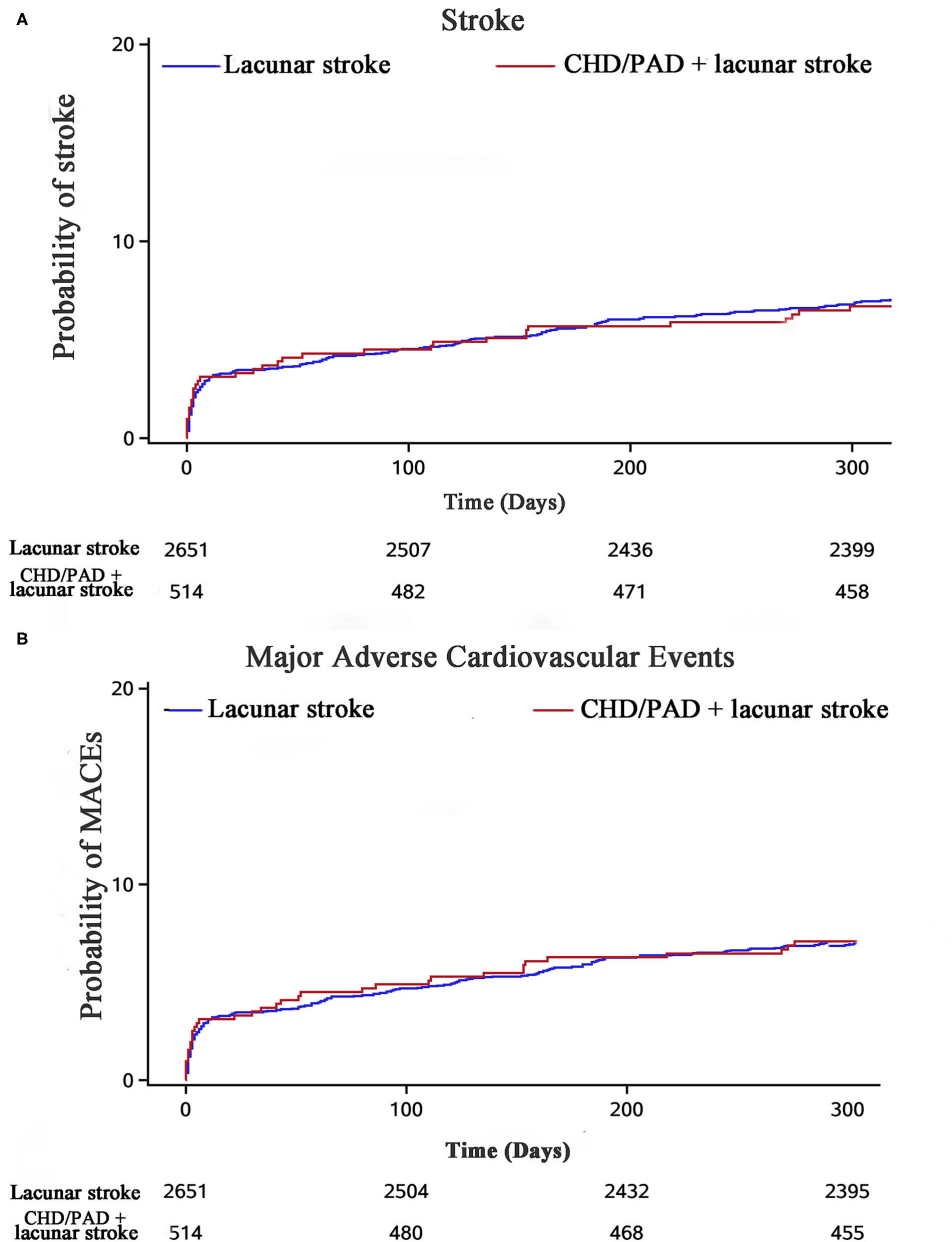
Stroke recurrence and MACEs in the study population stratified by polyvascular disease at 1 year.

During the 1-year follow up, stroke occurred similarly in patients with polyvascular disease. New stroke occurred in 27 (7.20%) patients with CHD and lacunar stroke, in 13 (7.74%) with PAD and lacunar stroke, in 36 (7.00%) with CHD/PAD and lacunar stroke, and in 198 (7.47%) with only lacunar stroke (Table 2). Kaplan–Meier curves showed that patients with polyvascular disease showed a similar occurrence of a new stroke at 1 year than those with damage to a single vascular bed (Figures 2, 3A).

**Table 2:** Outcomes in patients who were classified as having lacunar stroke at 1 year stratified by polyvascular disease.

	**Lacunar stroke alone (*N* = 2,651)**	**PAD and lacunar stroke (*N* = 168)**	**Hazard ratio (95%CI)**	***P-value***	**CHD and lacunar stroke (*N* = 375)**	**Hazard ratio (95%CI)**	***P-value***	**CHD/PAD and lacunar stroke (*N* = 514)**	**Hazard ratio (95%CI)**	***P-value***
Stroke recurrence	198 (7.47%)	13 (7.74%)	1.07 (0.61–1.87)	0.82	27 (7.20%)	0.98 (0.65–1.46)	0.91	36 (7.00%)	0.95 (0.66–1.35)	0.75
Ishchemic stroke	171 (6.45%)	13 (7.74%)	1.22 (0.70–2.14)	0.49	25 (6.67%)	1.01 (0.68–1.56)	0.89	34 (6.61%)	1.02 (0.71–1.48)	0.90
Hemorrhagic stroke	27 (1.02%)	0 (0)	0 (0)	0.98	2 (0.53%)	0.55 (0.13–2.33)	0.98	2 (0.39%)	0.39 (0.09–1.62)	0.19
MACE	205 (7.73%)	14 (8.33%)	1.11 (0.65-1.90)	0.71	29 (7.73%)	1.01 (0.69–1.49)	0.94	39 (7.59%)	0.99 (0.70–1.40)	0.95

*PAD, Peripheral Artery Disease; CHD, Coronary Heart Disease; MACEs, Major Adverse Cardiovascular Events; CHD/PAD, Coronary Heart Disease or Peripheral Artery Disease*.

Compared with patients with lacunar stroke alone, no interaction was observed for polyvascular disease and stroke recurrence, including CHD and lacunar stroke (HR, 0.98; 95% CI, 0.65–1.46; *p* = 0.91), PAD and lacunar stroke (HR, 1.07; 95% CI, 0.61–1.87; *p* = 0.82), and CHD/PAD and lacunar stroke (HR, 0.95; 95% CI, 0.66–1.35; *p* = 0.75). There was no significant association of polyvascular disease (for CHD: HR, 1.01; 95% CI, 0.68–1.56; *p* = 0.89; for PAD: HR, 1.22; 95% CI, 0.70–2.14; *p* = 0.49; for CHD/PAD: HR, 1.02; 95% CI, 0.71–1.48; *p* = 0.90) and the recurrence of ischemic stroke at 1 year in this study. The rate of hemorrhagic stroke was low in all groups of CHD and lacunar stroke (HR, 0.55; 95% CI, 0.13–2.33; *p* = 0.98), PAD and lacunar stroke (HR, 0; 95% CI, 0; *p* = 0.98), and CHD/PAD and lacunar stroke (HR, 0.39; 95% CI, 0.09–1.62; *p* = 0.19).

### Association Between Polyvascular Disease and MACEs

The proportion of MACEs in all four groups at 1 year is shown in Table 2. Among patients with MACEs, 29 (7.73%) had CHD and lacunar stroke, 14 (8.33%) had PAD and lacunar stroke, 39 (7.59%) had CHD/PAD and lacunar stroke, and 205 (7.73%) had lacunar stroke only.

Polyvascular disease was not associated with an increased risk of MACEs (Figure 3B). The HR (95% CI) of stroke recurrence/MACEs in patients with polyvascular disease is shown in Table 2. Rates of MACEs were similar for CHD and lacunar stroke (HR, 1.01; 95% CI, 0.69–1.49; *p* = 0.94), PAD and lacunar stroke (HR, 1.11; 95% CI, 0.65–1.90; *p* = 0.71), and CHD/PAD and lacunar stroke (HR, 0.99; 95% CI, 0.70–1.40; *p* = 0.95). There was no significant difference in the rate of MACEs in patients with polyvascular disease compared with patients with lacunar stroke alone.

## Discussion

We investigated the association between polyvascular disease and stroke recurrence/MACEs in patients with lacunar stroke. Among 3,165 patients with lacunar stroke, polyvascular disease (including CHD + lacunar stroke, PAD + lacunar stroke, and CHD/PAD + lacunar stroke) was not associated with an increased risk of stroke recurrence and MACEs compared with patients with only lacunar stroke.

Previous studies have shown that the risk of composite clinical outcomes increases with polyvascular disease compared with patients with only damage to a single vascular bed. The international Reduction of Atherothrombosis for Continued Health (REACH) Registry showed that the percentages of population of all-cause mortality and stroke recurrence were higher in patients with polyvascular disease compared with patients only with cerebral vascular disease (17). The EUCLID trial also showed that the risk of MACEs and lower extremity revascularization increased with polyvascular disease involvement compared with patients with PAD alone (5).

Our study is different from these previous studies (5, 17). Potential explanations for the differences between our study and other studies are as follows. We chose patients with recent lacunar stroke as the study population. The population in previous studies was patients with ischemic stroke. This suggests that the mechanism of lacunar stroke is different from that of large artery atherosclerosis. The pathological changes in lacunar stroke are arteriolosclerosis, lipohyalinosis, or fibrinoid necrosis of the small (40–200 μm in diameter) arterioles (18, 19). However, large artery atherosclerosis is due to fatty streaks, and fatty streaks gradually develop into atheroma and characteristic plaques. Acute rupture of these atheromatous plaques causes local thrombosis, leading to partial or total occlusion of the affected artery (1).

Our study had several limitations. First, all patients were Chinese in the CNSR-III, which may limit the generalizability of our findings to other populations. Second, we focused on CHD and PAD. Other types of damage to the vascular bed should be investigated in a future study. Additionally, we will further investigate the association between the subtype of stroke recurrence and polyvascular disease. The findings of this study should be evaluated further in large lacunar stroke cohorts and in different populations.

In conclusion, the presence of polyvascular disease is not associated with recurrence of stroke and MACEs in patients with recent lacunar stroke at 1 year.

## Data Availability Statement

The raw data supporting the conclusions of this article will be made available by the authors, without undue reservation.

## Ethics Statement

The studies involving human participants were reviewed and approved by The ethics committee of Beijing Tiantan Hospital. The patients/participants provided their written informed consent to participate in this study.

## Author Contributions

XM had full access to all of the data in the study and take responsibility for the integrity of the data and the accuracy of data analysis. LM, XM, YoW, and YiW: study concept and design. LM and XM: acquisition, analysis, or interpretation of data, and Drafting of the manuscript. YoW and YiW: critical revision of the manuscript and important intellectual contribution, obtained funding, and study supervision. AW and YZ: statistical analysis, and administrative, technical, or material support. All authors listed have made a substantial, direct and intellectual contribution to the work, and approved it for publication.

## Funding

This study was supported by grants from the Ministry of Science and Technology of the People's Republic of China (2016YFC0901001, 2016YFC0901002, 2017YFC1307900, 2017YFC1310901, 2018YFC1311700, and 2018YFC1311706), Beijing Municipal Committee of Science and Technology (D151100002015003, Beijing Excellent Talents Training and Supporting Top Youth Team, D171100003017001, and 2016000021223TD03), Beijing Municipal Commission of Health and Family Planning (Nos. 2016-1-2041 and SML20150502), the National Natural Science Foundation of China (81825007), Beijing Municipal Education Commission (Excellent Young Scientists Project), and the third batch of National Ten Thousand Talents Plan, and Beijing Outstanding Young Scientist Program (BJJWZYJH01201910025030).

## Conflict of Interest

The authors declare that the research was conducted in the absence of any commercial or financial relationships that could be construed as a potential conflict of interest.

## Publisher's Note

All claims expressed in this article are solely those of the authors and do not necessarily represent those of their affiliated organizations, or those of the publisher, the editors and the reviewers. Any product that may be evaluated in this article, or claim that may be made by its manufacturer, is not guaranteed or endorsed by the publisher.
